# Supplementary material for: Clinically Relevant Pancreatic Fistula after Pancreaticoduodenectomy: How We Do It
Source: Biology (Basel). 2023 Jan 22;12(2):178. doi: 10.3390/biology12020178 (PMC9952935; doi:10.3390/biology12020178)
Supplement: Supplementary file 1 [file biology-12-00178-s001.zip › biology-2076204-supplementary.pdf]

Table S1. Patients with clinically relevant PF °B/C- treatment strategies.

age (a)	sex (m/f)	CCI	Indication for pancreatectomy	POPF grade (B/C)	Treatment	Time of treatment (POD)	Further specification of treatment		Octreotide (yes/no)	Additional treatment with administration of jejunal tube for secondary DGE		Length of postoperative stay	Mortality (yes/no)
							Indication	Procedure		on POD	Total length (d)		
65	f	2	Distal bile duct cancer (cholangiocarinoma)	B	conservative	3		antibiotic treatment, successive removal of intraoperatively administered drainage	no	-	-	discharged on POD 26	no
53	f	0	Tumor of the major duodenal papilla (histological diagnosis: hyperplasia)	B	conservative	3		antibiotic treatment, successive removal of intraoperatively administered drainage	no	-	-	discharged on POD 30	no
70	m	3	Ductal adenocarcinoma of the pancreatic head	B	conservative	3		antibiotic treatment, successive removal of intraoperatively administered drainage	no	-	-	discharged on POD 22	yes; death 11 months after surgery for unknown reasons
55	m	2	Neurendocrine tumor (NET) of the major duodenal papilla	B	conservative	3		antibiotic treatment, successive removal of intraoperatively administered drainage	no	-	-	discharged on POD 34	no
59	m	0	High grade intraepithelial lesion of duodenum, mutation of MUTYH gen (histological diagnosis: high grade	B	conservative	3		antibiotic treatment, successive removal of intraoperatively administered drainage	no	-	-	discharged on POD 14 (with drainage; accidental removal of drainage on	no

			intraepithelial lesion of duodenum, PanIN 1b)								POD 40; no further associated complications)	
80	m	4	Ductal adenocarcinoma of the pancreatic head	B	conservative	3	antibiotic treatment, successive removal of intraoperatively administered drainage	no	14	11	discharged on POD 28	no
67	f	1	IPMN-MD (histological diagnosis: chronic pancreatitis)	B	conservative	3	antibiotic treatment, successive removal of intraoperatively administered drainage	yes	-	-	discharged on POD 22	no
30	m	2	Neuroendocrine tumor (NET) of Pancreas	B	conservative	3	antibiotic treatment, successive removal of intraoperatively administered drainage	yes	-	-	discharged on POD 22	yes; date and reason unknown
66	f	0	Tumor of the major duodenal papilla (histological diagnosis: adenoma)	B	conservative	3	antibiotic treatment, successive removal of intraoperatively administered drainage	yes	16	41	discharged on POD 89	no
67	m	3	Distal bile duct cancer (cholangiocarinoma)	B	conservative	3	antibiotic treatment, successive removal of intraoperatively administered drainage	yes	-	-	discharged on POD 25	yes; septic shock due to pulmonary sepsis on POD 114
65	f	0	IPMN-MD (histological diagnosis: IPMN-BD)	B	conservative	3	antibiotic treatment, successive removal of intraoperatively administered drainage	yes	14	11	discharged on POD 27	no
44	m	1	Malignant pancreatic tumor (histological diagnosis: chronic	B	conservative	3	antibiotic treatment, successive removal of	yes	12	1	discharged on POD 22	no

			pancreatitis with pseudocyst)					intraoperatively administered drainage				
75	m	2	Duodenal carcinoma with infiltration of pancreas	B	conservative	3		antibiotic treatment, successive removal of intraoperatively administered drainage	yes	-	-	discharged on POD 22	yes; died on POD 39 (resubmission POD 32, haemorrhagic shock due to arrosion bleeding A. hepatica)
43	f	0	Pancreatic head tumor (histological diagnosis: chronic pancreatitis)	B	conservative	3		antibiotic treatment, successive removal of intraoperatively administered drainage	yes	-	-	discharged on POD 13	no
73	f	4	Carcinoma of the major duodenal papilla (papillary carcinoma)	B	conservative	3		antibiotic treatment, successive removal of intraoperatively administered drainage	yes	11	16	discharged on POD 25	no
71	m	3	Ductal adenocarcinoma of the pancreatic head	B	drainage	10	retrogastral fluid collection close to PG	CT-guided drainage	no	-	-	discharged on POD 24	yes; death 6 months and 13 days after surgery due to liver abscesses and local recurrence of tumor with hepatic metastasis
35	m	0	Tryptische Nekrosen nach stumpfem Pankreastrauma	B	drainage	14	retrogastral fluid collection close to PG	CT-guided drainage (later additional transgastric drainage)	no	-	-	discharged on POD 41	no
68	m	9	Distal bile duct cancer (cholangiocarinoma)	B	drainage	17	retrogastral fluid collection close to PG	CT-guided drainage	yes	-	-	discharged on POD 26 (with CT-guided	no

												drainage; accidental removal of drainage on POD 40; no further associated complications)	
83	m	3	mucinous cystadenoma (histological diagnosis: IPMN-MD with high grade intraepithelial lesion)	B	drainage	4	fluid collection close to PG	CT-guided drainage, (later additional transgastric drainage)	yes	-	-	discharged on POD 34	no
66	m	4	IPMN-MD (histological diagnosis: IPMN-MD, PanIN 2)	B	drainage	12	fluid collection	CT-guided drainage, (later additional transgastric drainage)	no	-	-	discharged on POD 28	no
48	f	2	Ductal adenocarcinoma of the pancreatic head	B	drainage	6	retrogastral fluid collection	CT-guided drainage	yes	7	11	discharged on POD 30	no
75	m	2	Distal bile duct cancer (cholangiocarinoma)	B	drainage	14	retrogastral fluid collection	transgastral drainage	no	-	-	discharged on POD 27	no
59	m	1	mucinous cystic neoplasia (histological diagnosis: pseudocyst of the pancreas)	B	drainage	15	fluid collection	CT-guided drainage	yes	11	10	discharged on POD 35	no
81	f	0	perforation of duodenum after	B	drainage	12	retrogastral fluid collection	CT-guided drainage	yes	-	-	discharged on POD 34	no

			endoscopy (histological analysis: chronic pancreatitis)										
60	m	2	Distal bile duct cancer (cholangiocarinoma)	B	EVT	25	Insufficiency of PG	EVT 7 cycles	yes	-	-	discharged on POD 64	yes; death 2 years after sugery due to local recurrence of tumor with hepatic metastasis
65	m	2	Cystadenocarcinoma (histological diagnosis: chronic pancreatitis with pseudocyst)	B	EVT	2	Insufficiency of PG 10 mm	EVT 5 cycles	no	-	-	discharged on POD 36	
36	m	0	Chronic pancreatitis, opiod use	B	EVT	8	Insufficiency of PG 8 mm	EVT 3 cycles	yes	-	-	discharged on POD 31	no
54	m	3	Neurendocrine tumor (NET) of the major duodenal papilla	C	EVT	30	Insufficiency of PG 4 mm	EVT 3 cycles, restpancreatectomy due to severe bleeding on POD 15	no	-	-	discharged on POD 75	no
50	m	2	Distal bile duct cancer (cholangiocarinoma)	B	EVT	12	Insufficiency PG 10 mm	EVT 2 cycles	yes	24	6	discharged on POD 35	no
51	m	2	Neuroendocrine tumor (NET) of pancreas	B	EVT	26	Insufficiency PG 8 mm	EVT 2 cycles	no	-	-	discharged on POD 9, readmission on POD 23 with insufficiency of PG, then dischage on POD 35	no

78	m	2	Serous cystadenom (histological diagnosis: IPMN-BD)	C	surgery	12	Suspicious drainage fluid (small bowel leakage) and infarciation of the spleen	Splenectomy, lavage (no insufficiency found)	no	-	-	died on POD 15	yes; acute right heart failure and subsequent multiorgan failure on POD 15
73	m	2	Cystic tumor of pancreatic head, rapidly increasing in size (histological diagnosis: IPMN-BD)	C	surgery	3	dehiscence of abdominal wound	lavage and additional instillation of saline through intraoperative adminstered drain	yes	-	-	discharged on POD 86	no
81	m	8	Ductal adenocarcinoma of the pancreatic head	C	surgery	POD 13	arrosion bleeding A. hepatica communis	completion pancreatectomy and splenectomy	yes	-	-	discharged on POD 43	no
79	m	4	Ductal adenocarcinoma of the pancreatic head	C	a) surgery<br>b) drainage<br>c) EVT	a) 1 + 10<br>b) 11<br>c) 22	a) bleeding (splenic artery) and removal of haematoma<br>b) fluid collection<br>c) Insufficiency PG 15 mm	a) reinsertion pancreas<br>b) CT-guided drainage<br>c) EVT 6 cycles	no	-	-	died on POD 135	yes; septic shock due to pulmonary and urogenital sepsis
43	m	0	Familial adenomatous polyposis with duodenal polyposis (histological	C	a) drainage<br>b) EVT	a) 14<br>b) 15	a) retrogastral fluid collection<br>b) Insufficiency PG 8 mm	a) CT-guided drainage<br>b) EVT 11 cycles	yes	-	-	discharged on POD 70	no

			diagnosis: multiple adenoma of duodenum with high grade intraepithelial lesions)										
58	m	5	Carcinoma of the major duodenal papilla (papillary carcinoma)	C	a) EVT<br>b) drainage	a) 12<br>b) 16	a) Insufficiency PG 6 mm<br>b) retrogastral fluid collection	a) EVT 1 cycle<br>b) CT-guided drainage	yes	-	-	died on POD 18	yes; acute arrosion bleeding
65	m	2	Distal bile duct cancer (histological diagnosis: chronic pancreatitis)	C	a) surgery<br>b) EVT	a) POD 1<br>b) POD 12	a) drainage with airleak<br>b) partial insufficiency PG	a) reinsertion of pancreas<br>b) EVT 1 cycle	yes	-	-	died on POD 14 due to haemorrhagic shock due toarrosion bleeding A. hepatica	yes
54	m	2	Carcinoma of the major duodenal papilla (Papillary carcinoma)	C	a) EVT<br>b) surgery<br>c) EVT	a) POD 7<br>b) POD 10<br>c) POD 13	a) insufficiency PG 8 mm<br>b) partial dehiscence PG<br>c) re-insufficiency PG	a) EVT 1 cycle<br>b) reinsertion of pancreas<br>c) EVT 3 cycles	yes	-	-	discharged on POD 34	no
61	f	2	Pancreatic head tumor (histological diagnosis: IPMN-BD)	C	a) surgery<br>b) EVT	a) POD 1<br>b) POD 4	a) partial dehiscence PG<br>b) insufficiency PG	a) reinsertion of pancreas<br>b) EVT 7 cycles	yes	-	-	discharged on POD 40	no

EVT = endoscopic vacuum assisted therapy, IPMN-BD = intraductal papillary mucinous neoplasia of branch duct, IPMN-MD = intraductal papillary mucinous neoplasia of main duct, PanIN = pancreatic intraepithelial neoplasia, POPF = postoperative pancreatic fistula, POD = postoperative day
